# Assessing Appetite–Validation of a Picture-Based Appetite Assessment Tool for Children Aged 6–9 Years—A Pilot Study

**DOI:** 10.3390/nu17213347

**Published:** 2025-10-24

**Authors:** Sigal Eilat-Adar, Yoav Zeevi, Efrat Shaked, Yael Rabih, Sima Zach

**Affiliations:** 1 Levinsky-Wingate Academic College, Wingate Campus, Post Graduate Department, Netanya 4290200, Israelsimaz@l-w.ac.il (S.Z.); 2 Independent Researcher, Tel Aviv 6291080, Israel; yaelcr@outlook.com

**Keywords:** Picture-Based Appetite Assessment tool, hunger, satiety, children aged 6–9 years

## Abstract

**Background:** Recognizing and balancing internal and external appetite cues is critical for controlling food intake in young children. The main aim of this pilot study was to validate a Hebrew-language Picture-Based Appetite Assessment (PBAA) for 6–9-years-old children. Specifically, the scale’s ability to reflect changes in perceptions of hunger and satiety based on a story and on their actual eating experiences. **Methodology:** In Part 1 (*n* = 99), a PBAA was used to rate a character’s hunger level according to a story. In Part 2 (*n* = 46), the child’s hunger level before and after lunch was assessed, and in Part 3 (*n* = 55), the child’s hunger level before and after unrestricted snack consumption was assessed. **Results:** After hearing a story, participants could identify whether a character in a story was hungry (95%) or full (85%) (Part 1). Their reported appetite levels decreased after consuming lunch (*p*-value < 0.001) (Part 2). When participants were given unrestricted access to snacks, they preferred highly processed sweets with no difference in hunger level before (Part 3). There were no differences between girls and boys except for the reported satiety after lunch, which was greater in older girls compared to younger girls, yet similar between older and younger boys. **Conclusions:** Participants successfully interpreted the PBAA scale based on the story character and reported lower hunger after eating lunch. Among girls, older age was associated with a greater difference in hunger levels before and after lunch. Most participants reported satiety after consuming unrestricted snacks, which was not related to their hunger level before.

## 1. Introduction

Overweight and obesity in children are defined in two primary ways: The first way relies on the body mass index (BMI) for age, where BMI values ≥ the 85th and 95th percentiles indicate overweight and obesity, respectively [1]. In the second way, used by the World Health Organization (WHO) [2], overweight and obesity in children are calculated using weight-for-height z-scores: values above two standard deviations (SD) from the median of the WHO growth standards indicate overweight, while values above three standard deviations indicate obesity. On the other hand, children with values above (−2) SD are considered wasted, and those with values between (−3) SD are considered malnourished. WHO data highlight a dramatic increase in the prevalence of overweight and obesity among children and adolescents aged 5–19, rising from just 4% in 1975 to over 18% in 2016, with no significant difference between boys and girls [3]. Over 390 million children and adolescents aged 5–19 years were overweight in 2022, including 160 million who were living with obesity [4]. To address the growing prevalence of childhood obesity, global efforts are focused on developing and implementing educational, medical, and public health interventions [5,6].

The development of obesity in children involves physical and psychological factors that influence energy balance, appetite regulation, and energy intake [7]. As children grow older, their ability to self-regulate is increasingly influenced by external factors, such as food availability, portion size, palatability, and parental behaviors. These environmental influences may shift children’s attention from internal cues to external ones, impairing self-regulation, which increases the risk of obesity [7]. All these factors are lacking in children with malnutrition and nearly all of them report reduced appetite.

Recognizing and balancing internal and external appetite cues is critical for controlling food intake and maintaining a healthy weight and growth trajectory in children [8]. Early-life interventions are essential, as eating habits established during childhood are likely to persist into adolescence and adulthood [9,10].

This evidence highlights the need for tools that can assess appetite and support interventions aimed at fostering mindful eating habits from a young age.

Feelings of hunger and satiety are typically measured using a **Visual Analogue Scale (VAS)**, a subjective tool used to predict food intake. However, the VAS is generally used for children aged 8 years and older [11,12].

Another common approach to assessing appetite is parental reporting using tools like the **Children’s Eating Behavior Questionnaire (CEBQ)** [13], which assesses the child’s responsiveness to food, emotional overeating, and enjoyment of eating. However, parental reporting has one main limitation, which is the reporting bias inherent in parental perceptions of the child’s eating behavior. This perception is influenced by factors such as parental feeding styles, which may lead to a report that does not completely reflect the true extent of the children’s feelings of hunger and satiety. A scientific evaluation of the Portuguese version [14], of the CEBQ found that parental reports are influenced by confounding factors such as lifestyle, family income, parental age and education, family structure, limiting the accuracy of the child’s true hunger and satiety perceptions. Likert scales with five ratings, often in pictorial formats, are considered more suitable for young children [15]. Scales developed for children aged 3–6 years often feature cartoon characters with varying amounts of food in their stomachs, an area intuitively linked to hunger and satiety [16].

Recently, the **Picture-Based Appetite Assessment (PBAA)** tool was validated for children aged 4–10 years to measure appetite both in states of hunger and after eating [17]. The PBAA was found to be reliable, as children reported different appetite levels before and after consuming unrestricted snack portions. However, some PBAA validation studies excluded the responses of children who provided unexpected satiety ratings, without ascertaining whether these children understood the scale [18]. Identifying the gap in knowledge between scientific data and clinical practice is needed to formulate evidence-based recommendations that may potentially be used not only to prevent obesity but also to evaluate malnutrition [19]

In the absence of appetite assessment scales for children in Hebrew, a novel image-based rating scale was developed (Appendix A). This scale consists of five illustrated characters (with separate scales for boys and girls), each depicting varying amounts of “food” represented by a circle in the center of the stomach, alongside a verbal description of each character’s level of appetite. The purpose of the scale is to enable children to express their feelings of hunger or satiety. It was designed to introduce children to the principles of healthy nutrition through dialogue and play. The toolkit addresses topics such as non-physiological eating, eating ‘when you feel like it,’ overeating, and more. The “Appetite Lexicon” was developed by Yael Chen Rabia, a senior clinical dietitian, in collaboration with Sharon Tal, a child psychologist and educational content developer. The toolkit is suitable for children from kindergarten age (with parental guidance) and has received a recommendation from the Adler Institute, Israel [20].

### 1.1. Main Objective

The primary goal of this pilot study was to assess whether children aged 6–9 years old understand a Hebrew-language PBAA tool. Specifically, the study aimed to assess the scale’s ability to reflect changes in perceptions of hunger and satiety based on storytelling and feelings of hunger and satiety based on their actual eating experiences.

### 1.2. Secondary Objectives

1. To determine whether participants can accurately rate hunger levels that correspond to the hunger levels of the child in a story (Part 1)
2. To evaluate the correlation between post-lunch hunger levels and ratings on the PBAA scale (Part 2)
3. To assess the alignment between hunger levels after lunch, using the PBAA rating and the type of food, and calorie intake of self-selected snacks (measurable by the researchers) (Part 3)
4. To assess the alignment between hunger levels using the PBAA rating and the type of food, and calorie intake after eating self-selected snacks (measurable by the researchers) (Part 3)
5. To verify whether participants who report higher or no change in hunger levels after meals (Parts 2 and 3) genuinely feel as indicated by their responses
6. To examine potential sex and age differences in scale comprehension

### 1.3. Research Hypotheses

1. Participants will rate hunger levels in alignment with hunger and satiety descriptions in the story.
2. Participants will report lower hunger levels after eating lunch.
3. Participants will report lower hunger levels after consuming self-selected snack portions (measurable by the researcher).
4. Participants who will not report lower hunger levels after meals (Parts 2 and 3) will genuinely feel as indicated by their responses
5. There will be no significant sex or age differences in scale comprehension.

## 2. Methods

This was an observational cross-sectional pilot study employing a replication and extension approach based on the work of Bennett and Blissett [18], who approved its replication.

### 2.1. Study Population

The study enrolled 154 boys and girls aged 6–9, all native Hebrew speakers, including 51 participants in Part 1, 48 participants in Part 2, and 55 participants in Part 3.

This was a convenience sample drawn from all the children attending municipal after-school programs operated by the Israel Association of Community Centers in the towns of Yokneam (2023) and Mevasseret Zion (2024). Both towns have high socioeconomic statuses (index 7–8 out of 10 in the Central Bureau of Statistics, 2024 [21]. This age group (Grades 1–3) was chosen based on the assumption that at these ages children are able to read the verbal explanations accompanying each character illustrated on the scale. The sample size was determined based on a similar study by Jacqueline Blissett, on which this study is based, in which 47 children were involved in each phase [18].

Exclusion Criteria: Children with food allergies, food sensitivities, cognitive development delays, or chronic metabolic conditions such as diabetes or celiac disease.

### 2.2. Research Procedure

The study was conducted in accordance with the Declaration of Helsinki. After obtaining approvals from the after-school program coordinators, the protocol was approved by the Levinsky-Wingate Institutional Ethics Committee #376, on 27 November 2022. Parents signed informed consent forms and completed a short questionnaire, which was coded.

### 2.3. Research Parts

#### 2.3.1. Preliminary Sessions

Children participated in two sessions to familiarize themselves with the “Appetite Lexicon” toolkit and its included scale.

**Part 1: Story-based satiety assessment**

1. Fifty-one children met individually with one of 2 study researchers, for 10 min in a quiet space in the community center. The PBAA scale was presented, and the researcher confirmed comprehension by asking “How hungry or full does this character seem to you?” while pointing to each character on the scale. Accuracy in identifying the character’s satiety level served as a gold standard for validating the scale’s concurrent validity (Appendix A), as the children were expected to experience greater satiety after the meal. Such responses would serve as concurrent validation of the research tool.

The researcher read a story to the child in which the hunger level of a character was clearly described (Appendix B—the story in English, Appendix C—the story in Hebrew). The child was then asked to rate the hunger or satiety of the story’s character before and after a meal. Correspondence between what the child said and what they indicated on the scale was examined.

**Part 2: Lunch-based satiety assessment**

1. Forty-eight children were assessed upon arriving at the after-school program in the community center and before consuming lunch. The scale and its ratings were explained to the children.
2. The researcher read a story to the children, and they were then asked to indicate the level of hunger or satiety of the character in the story as well as their own hunger levels using the scale. For the story comprehension analysis, data from the two parts, 1 and 2, were combined. Because the story component, instructions, and scoring procedures were identical and the samples were mutually exclusive, pooling was appropriate to strengthen the validity assessment while maintaining independent observations.
3. Lunch was offered for all the children at the same time, and was identical for all of them (fixed menu of the municipal after-school program for children aged 6–9).
4. Post-lunch, children rated their hunger levels again on the scale. If a child rated their hunger like or higher than their ratings before eating, the researcher ensured the child understood the question and verified that the response reflected the child’s true feelings.

The repeated exposure to the PBAA scale and story tasks reflects the tool’s intended educational use rather than a learning effect. All participants followed a standardized procedure under identical conditions, minimizing potential priming or order effects

**Part 3: Snack-based satiety assessment**

1. Fifty-five children met individually with the researcher after lunch at the community center. The researcher introduced the PBAA scale to the participants and confirmed their comprehension by asking them “How hungry or sated does the character in the scale seem to be?” for each character.
2. Children rated their own hunger levels using the scale.
3. Each child was offered a selection of 200 g of apple slices, 200 g of carrot sticks, 200 g of meringue candies, 150 g of jam-filled cookies, 70 g of rice cakes, and 80 g of pretzel sticks (snack options considered to be minimally harmful).
4. The child was left alone with the snacks for 10 min, while the researcher stood behind the door, during which time they had been told they could eat freely.
5. After 10 min, the researcher returned, removed the snacks, discussed the child’s snack preferences, and two minutes later asked them to rate their hunger again on the scale. If the child rated their hunger similar to or higher than before eating, the researcher ensured they understood the question.
6. The association between hunger levels before and after eating snacks was measured according to calories consumed, and food preferences were assessed.

**Figure 1:**
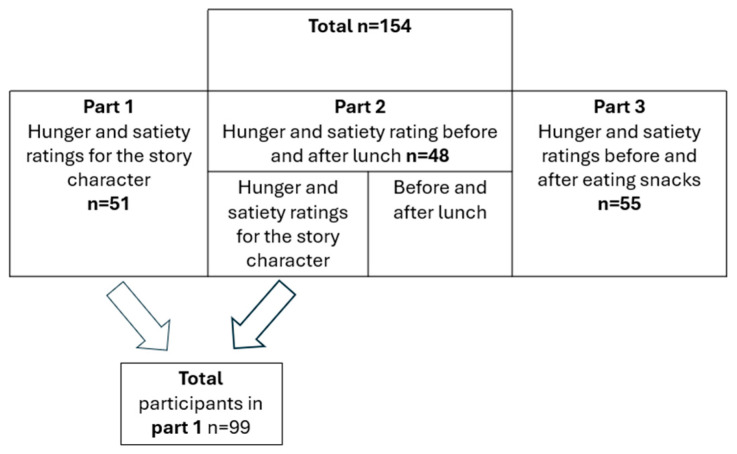
Chart flow of participants in the study.

Participants received a magnet featuring the hunger-satiety scale as a thank-you gift. The remaining snacks were weighed to calculate the calories consumed, as described under Data analysis. Figure 1 summarizes the number of participants in each part of the study

#### 2.3.2. Research Tools

- A rating scale containing pictures of boys (for the boys) or girls (for the girls), comprising a series of five illustrations depicting children with varying amounts of food in their “belly.” The pictures show differently colored children, each with a circle filled to a different level (representing the amount of food) located at the center of their body. These visualizations aim to represent feelings of hunger, ranging from 5 (“very very hungry”) to 1 (“stuffed”). The facial expressions of the illustrated children vary to reflect their hunger levels, and each figure is accompanied by a written description of their hunger levels in Hebrew for children who can read.
- Parent questionnaire.
- Narrative about a child in different states of hunger.
- Measured the quantity of snacks.

Research instructions were provided to the researchers prior to the experiment

### 2.4. Data Analysis

We used the Journal Usage Statistics Portal (JASP) statistical software, version 20 for data analysis. Non-parametric tests were employed due to the ordinal nature of the variables, as no assumption of normal distribution could be made.

- A Wilcoxon signed rank test was applied for dependent samples to assess whether a significant difference existed in children’s ratings of the hunger/satiety levels of a character in the story at two points in time—before and after a meal (Part 1). In Part 2, a Wilcoxon signed rank test was implemented to examine differences in each child’s personal hunger ratings pre- and post- lunch.
- A Mann-Whitney U test for independent samples was used to evaluate whether sex influenced children’s ratings of hunger for the character in the story, both pre- and post-meal, as well as their own hunger perceptions before and after a meal.
- Spearman rank correlation [ρ] analyses were conducted to determine whether a child’s age impacted their accuracy in assessing the hunger levels of the character before and after a meal, as well as their self-reported hunger levels before and following lunch. No correction for multiple comparisons was applied, consistent with the pilot design and the limited number of planned comparisons.

In Part 3, the number of consumed snacks was converted into caloric intake using the USDA’s calorie calculation software, version 13.2. To assess whether changes in reported hunger were observed on the scale in response to snack consumption under controlled conditions, a Wilcoxon signed-rank test was conducted. Additionally, the relationship between reported hunger levels and the caloric amount of snacks consumed was evaluated by Spearman’s correlations.

## 3. Results

### 3.1. Part 1: Hunger and Satiety Ratings for the Story Character

In total, data from 99 children (49 boys [45.5%] and 50 girls [55.5%]) were analyzed. The mean age (years) was 7.00 (SD = 0.75 years), with no significant difference between boys and girls (*p*-value = 0.29).

The response considered correct for rating the hunger level of the story character before a meal was 5, while the response considered correct for after a meal was 1. Table 1 presents the median (Mdn) and interquartile range (IQR) of responses, the number and percentage of children who answered correctly at the beginning and end of the story, and statistical comparisons of ratings for the story characters before and after the meal using the Wilcoxon signed rank test. Data are displayed as *n* (%) for categorical variables and as Mdn and IQR for continuous variables.

The majority of children accurately rated the hungerlevels of the story character: 5 before the meal and 1 after the meal. When ratings 4 before the meal and 2 after were also considered correct, only one boy (approximately 1%) answered incorrectly for the pre-meal level, and two children gave incorrect responses for the post-meal condition. Statistical testing was not performed for the girls’ pre-meal ratings due to the lack of variance (all girls responded identically).

A Mann-Whitney U test comparing the response accuracy between boys and girls revealed no significant difference based on sex (*p*-value = 0.56) or age (*p*-value = 0.09). However, when comparing the response accuracy between younger and older children, there was a trend suggesting younger children were more likely to provide incorrect responses (*p*-value = 0.09).

**Figure 2:**
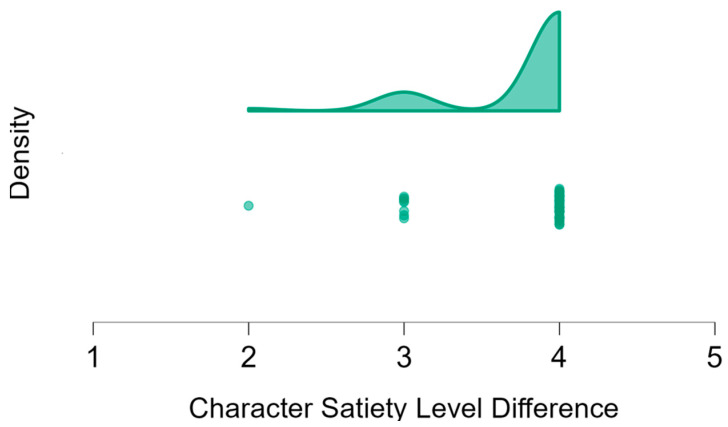
The difference in hunger levels of the character in the story. A higher number represents the difference in the character’s hunger at the beginning vs. the end of the story. As presented, most of the children reported the highest difference in hunger. The dots represent the frequency of children’s satiety levels.

Figure 2 illustrates a dot plot frequency and a raincloud frequency distribution of the differences in hunger ratings of the character before and after the story as rated by all 99 participants (Parts 1 and 2).

### 3.2. Part 2: Hunger and Satiety Rating Before and After Lunch

Part 2 included 48 participants. However, as data from two participants were missing, they were excluded from the data analysis, resulting in 46 children (20 boys and 26 girls). The mean age ± SD (years) of the entire group was 7.0 ± 0.74, with no significant difference between boys and girls (*p*-value = 0.12).

Table 2 presents the children’s responses to the question regarding their hunger level before and after lunch. The data are reported as *n* (%) for categorical variables and as Mdn and IQR for continuous variables.

Children reported feeling “hungry” (5) before lunch and “full” (1) after lunch, and the differences observed were statistically significant (*p*-value <0.001 for all participants, boys only, and girls only; Table 2). A Spearman’s correlation test revealed a significant positive correlation between age and the change in hunger levels before and after the meal (ρ = 0.40, *p*-value = 0.002). Older children tended to report greater satiety after the meal. Of note, however, this association was statistically significant only among girls (ρ = 0.60, *p*-value = 0.001).

**Figure 3:**
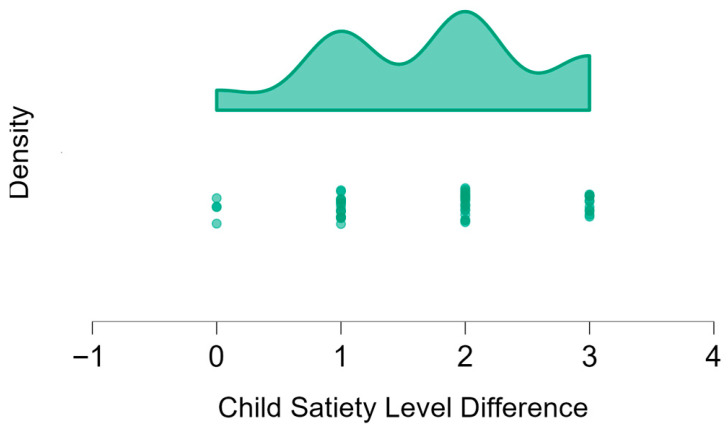
The difference in hunger levels of the child (participant) before and after lunch. Raincloud and dot plots of the difference in own hunger levels of the children before and after lunch, as rated by participants in Part 2 (*n* = 46). A higher number represents the difference in the child’s hunger at the beginning vs. the end of the meal. As presented, most of the children reported the highest difference in hunger. Zero represents no change, and minus represents a greater hunger of the character after the meal. The dots represent the frequency of children’s satiety levels.

Figure 3 displays the distribution of the change in own hunger ratings before and after lunch for the entire group of Part 2 participants, presented as raincloud and dot plots.

### 3.3. Part 3: Hunger and Satiety Ratings Before and After Eating Snacks

Fifty-five children with a mean ± SD age (years) of 7.00 ± 0.74 participated in this part. The 25 boys were on average 6.88 ± 0.60 years old, while the 30 girls were on average 7.23 ± 0.63 years old. Table 3 presents the ratings of the children regarding their own hunger levels before and after eating an unlimited number of snacks.

A statistically significant difference was found between the hunger rating before and after eating the snacks in all participants, as well as in boys only and girls only (Table 3). All but four children rated themselves as less hungry after eating the snacks (individual data not shown in Table 3).

**Figure 4:**
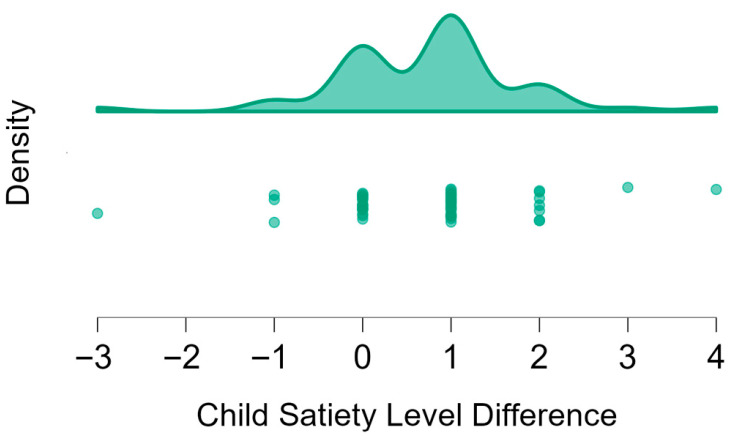
The difference in hunger levels before and after eating snacks. A higher number represents the difference in the child’s hunger at the beginning vs. the end of the meal. As presented, most of the children reported the highest difference in hunger. Zero represents no change, and minus represents a greater hunger of the character after the meal. The dots represent the frequency of children’s satiety levels.

Figure 4 displays the distribution of the change in own hunger ratings before and after eating the snacks, presented as raincloud and dot plots. Positive values indicate reduced hunger (*n* = 55).

Approximately one-third of the children (17, 30.91%) reported that their sensation of hunger remained unchanged after consuming the snacks. The majority of participants (34, 61.81%) indicated that they felt more sated following snack consumption, with only four children reporting feeling hungrier after consuming the snacks. When asked to elaborate, the four children explained that the snacks increased their desire to eat more.

A Mann-Whitney test revealed no statistically significant difference between boys and girls regarding the hunger sensation before (*p*-value = 0.31) and after (*p*-value = 0.06) eating snacks. However, although the findings did not reach statistical significance, the results do point towards girls feeling less satiated after compared to boys, despite no initial differences between the two groups prior to the meal. Table 4 presents the mean ± SD amount (in grams) consumed of each snack and the total caloric intake (in Kcal).

The greatest amount (grams) of snacks consumed was of the sweetest foods, namely, jam-filled cookies and breakfast cereals, followed by salty sticks (Table 4). Conversely, the children consumed minimal amounts of grams of carrots and apples, indicating a preference for the most highly processed foods.

A Spearman correlation test revealed a significant relationship between caloric intake and the change in hunger levels before and after the meal (ρ = 0.32, *p*-value = 0.02). Specifically, the more calories a child consumed, the greater the positive difference between their hunger levels before and after consuming the snacks. However, no significant correlation was found between pre-snack hunger levels and caloric intake (ρ = 0.18, *p*-value = 0.21). A weak but significant correlation was identified between caloric intake and post-snack hunger levels (ρ = 0.29, *p*-value = 0.04). No correlation was observed between age and caloric intake (ρ = −0.13, *p*-value = 0.35). Additionally, an independent *t*-test showed no significant association between sex and caloric intake (t (49) = 0.41, *p*-value = 0.69).

## 4. Discussion

As VAS has been deemed unreliable in younger children. In the current pilot study, parts 1 and 2 aimed to assess whether children aged 6–9 years old understand a Hebrew-language PBAA tool, i.e., to validate a culturally adapted PBAA tool for children aged 6–9, accounting for age and sex differences. We also explored the reasons behind children reporting hunger differently than expected. Previous studies did not explore this angle [18]. Our study revealed that after hearing a story in which the hunger level of a character was clearly described, children could identify whether a character in the story was hungry or full. This means that this small sample of children understood the PBAA. As we expected, most children ate their after-school lunch until they were less hungry. Similar findings were reported in a study involving 15 children aged 4–10 years, indicating that the PBAA tool can effectively detect expected changes in appetite sensations [17]. A study in 17 older children aged 9–14 years evaluated hunger levels before an ad libitum lunch lasting 35 min, comparing a hard copy (printed) VAS with a novel digital VAS, with and without images. In this age group, statistically significant correlations were observed between food intake and hunger across all three instruments, demonstrating good agreement [22].

A higher hunger level after lunch may indicate that the child didn’t understand the index, or that he didn’t like or couldn’t eat the food. In this case, the information is crucial for adjusting the food to the children’s palatability. On the other hand, this may represent a detachment of satiety and hunger feeling, which may need further intervention. In the current study,

In Part 3 of our study, we assessed the alignment between hunger levels using the PBAA rating and the type of food, as well as calorie intake after consuming self-selected snack portions (measurable by the researchers). When children who had a greater appetite after snack consumption were asked to elaborate, they explained that the snacks increased their desire to eat more, showing that they understood the index.

Children were allowed to eat an unlimited amount of selected ultra-processed and unprocessed foods. Except for four children, all participants reported lower hunger levels after eating snacks. Notably, their hunger levels had already been lower after lunch compared to before lunch. The highest caloric intake was observed from the sweetest foods, specifically jam-filled cookies and breakfast cereals. This aligns with characteristics of ultra-processed foods: “The processes and ingredients involved in its production are tailored to create products that are highly profitable (using low-cost ingredients, ensuring a long shelf life, and leveraging prominent branding), convenient (ready-to-eat), and hyper-palatable, often replacing unprocessed or minimally processed foods” [23].

A cross-sectional study of 1291 children aged 4–16 revealed that participants rated fatty and sugary foods most appealing across ages and sexes, although certain fruits also received high ratings [24]. Highest preferences were for chocolate, pizza, ice cream, pasta, strawberries, chocolate biscuits, ice lollies, grapes, cakes, and fruit sweets. Vegetables and certain meats and meat substitutes were consistently among the least preferred foods. In this study, sex differences in specific preferences were significant: boys more than girls favored fatty and sugary foods (*p* < 0.005), meat (*p* < 0.001), processed meat products (*p* < 0.001), and eggs (*p* < 0.05) more than girls did [24]. Physiological, psychological, and sociocultural factors may condition differences between genders and diet [25]. In our study, girls tended to report lower satiety after lunch. Assessing the diversity of food eaten at lunch may reveal the reason for this difference. A smaller change in hunger levels before and after lunch was observed in younger children, potentially reflecting a developmental trend. Despite the observed association between caloric intake and a larger change in hunger levels before and after the meal, no correlation was found between hunger levels before snack consumption and calories consumed. The findings of part 3 underscore the importance of educating children about healthy eating and the role of ultra-processed foods.

This pilot study has several limitations: It included a single assessment conducted immediately after the meal. The amount of food consumed at lunch was not measured, and children’s weight, height, and Z-scores were not calculated. Furthermore, the sample size was small. Low SES significantly impacts children’s ability to understand learned material through multiple pathways, including access to educational resources, family support, and cognitive and language development opportunities [26]. Therefore, the current study, which is implemented only in high SES children, has low external validity. Due to the limited sample size, no correction for multiple comparisons was applied. Future studies should validate the tool in other SES and larger populations, which can enable test-retest reliability or internal consistency. A larger sample size will allow us to include additional variables, such as anthropometric data and dietary intake, that will be adjusted for.

## 5. Conclusions

Our pilot study suggests that children aged 6–9 years old understand a Hebrew-language-adapted PBAA tool. It also suggests that using the scale may reflect changes in perceptions of hunger and satiety, based on both storytelling and actual eating experiences. In this perspective the tool was validated. The tool was used to assess hunger level according to amount and type of snack preferences. Children preferred snacks and ultra-processed foods over vegetables and fruits, with no association with their hunger level after lunch. After validation in younger ages and more socioeconomically and ethnically diverse populations, the PBAA tool can be used in developing and evaluating programs for conscious eating based on internal cue rather than external cue, at a younger age. It will be possible to expand its use in special populations, such as children with a lack of body weight regulation or with a developmental disability.

## Figures and Tables

**Table 1:** Hunger ratings of the story character, by sex.

Participants	*n* (%)			Mdn (IQR)		Before/After *	*p*-Value
	All	Rated 5 Before Meal	Rated 1 After Meal	Before Meal	After Meal		
**All**	99 (100)	96 (97.0)	84 (84.8)	5 (0)	1 (0)	8.55	<0.001
**Boys**	49 (45.5)	46 (93.9)	41 (83.7)	5 (0)	1 (0)	5.97	<0.001
**Girls**	50 (55.5)	50 (100.0)	43 (86.0)	5 (0)	1 (0)	**	

IQR = interquartile range; Mdn = median. ** The variance in “Child Before equals 0. * The Before/after comparison was performed using the Wilcoxon signed rank test.

**Table 2:** Hunger and satiety levels before and after lunch (a higher grade represents a higher hunger level), as rated by the participants of Part 2 (*n* = 46).

Participants	*n* (%)	Rating Before Lunch Mean ± SD	Rating After Lunch Mean ± SD	Mdn (IQR)		Z *	*p*-Value
				Before Lunch	After Lunch		
**All**	46(100)	4.02 ± 0.33	2.20 ± 0.89	4 (1)	2 (0)	5.71	<0.001
**Boys**	20(43.5)	4.10 ± 0.31	1.90 ± 0.91	4 (1.3)	2 (0)	3.82	<0.001
**Girls**	26(56.5)	3.96 ± 0.34	2.42 ± 0.81	4 (1)	2 (0)	4.27	<0.001

IQR = interquartile range; Mdn = median; SD = standard deviation. * As calculated by Wilcoxon test.

**Table 3:** Hunger and satiety levels before and after eating an unlimited number of snacks, as rated by participants in Part 3 (*n* = 55).

Participants	*n* (%)	Rating Pre-Snacks Mean ± SD	Rating After Snacks Mean ± SD	Mdn (IQR)		Z *	*p*-Value
				Before	After		
**All**	55(100)	4.04 ± 0.69	3.31 ± 0.84	4 (1)	3 (1)	4.21	<0.001
**Boys**	25(45.5)	3.96 ± 0.94	3.40 ± 0.77	4 (1)	4 (0)	2.22	0.019
**Girls**	30(55.5)	4.10 ± 0.40	3.20 ± 0.89	4 (1)	3 (0)	3.72	<0.001

IQR = interquartile range; Mdn = median; SD = standard deviation. * As calculated by Wilcoxon test.

**Table 4:** Mean and SD grams consumed per snack and grams of total caloric intake (*n* = 55).

Statistical Parameter	Snack Type (Grams)						Total Caloric Intake (Kcal)
	Apples	Carrots	Breakfast Cereals	Jam-Filled Cookies	Rice Crispies	Salty Sticks	
**Mean**	5.5	2.7	68.7	93.7	25.1	32.2	233.4
**SD**	11.5	5.3	142.7	110.6	33	46.2	197.2

Kcal = kilocalories; SD = standard deviation.

## Data Availability

Data are available upon request from the corresponding author.

## Abbreviations

PBAA	Picture-Based Appetite Assessment
BMI	Body Mass Index
WHO	World Health Organization
SD	Standard Deviation
VAS	Visual Analogue Scale
CEBQ	Children’s Eating Behavior Questionnaire
JASP	Journal Usage Statistics Portal
ρ	Spearman rank correlation
Mdn	Median
IQR	Interquartile range

## Appendix B. The Story in English Version

Story About Different Levels of Hunger and Fullness

(Danny is used for the boys; Dana is used for the girls)

Last Sunday, Danny/Dana went to the park to play on the equipment with friends. Danny/Dana spent the entire morning running around and playing. As time passed, Danny/Dana began feeling very hungry since it had been a long time since breakfast. His/Her tummy started to “rumble,” so they hurried home to eat lunch.

On the way, he/she thought about the food he/she would love to eat (ask children for their preferences). When Danny/Dana got home, lunch was waiting: bean soup, schnitzel, peas with potatoes, and a vegetable salad. He/She sat down to eat.

After finishing the soup, Danny/Dana felt he/she was no longer hungry but still had room to eat more. Mom served the main course, Danny/Dana finished everything, and smiled with satisfaction.

After the meal, he/she opened the cupboard and ate an entire bag of rice cakes, a lot of chocolate biscuits, and a glass of juice. His/Her tummy was so full and he/she felt tired. He/She was definitely not hungry anymore! (Ask children to share where Danny/Dana would rank on the hunger/fullness scale.)

## Appendix C. The Story in Hebrew

ביום ראשון שעבר, דני/דנה הלך/ה לגינה לשחק במתקנים עם החברים. דני/דנה בילה/תה את כל הבוקר בריצה בגינה ובמשחק. ככל שעבר הזמן דני/דנה התחיל/ה להרגיש מאוד רעב/ה, עבר הרבה זמן מאז שאכל/ה את ארוחת הבוקר שלו/ה. בטנו/ה של דני/דנה “קרקרה” והוא/היא מיהר/ה לחזור הביתה לאכול את ארוחת הצהריים שלו/ה. בדרך הביתה חשב/ה על האוכל שהוא/היא ישמח/תשמח לאכול (דירוג ילדים). בבית ציפתה לדני/דנה ארוחת הצהריים. מרק שעועית, שניצל, אפונה עם תפוחי אדמה וסלט ירקות. דני/דנה ישב/ה לאכול. אחרי שאכל את המרק הוא/היא הרגיש/ה שהוא/שהיא כבר לא רעב/ה אבל שיש לו/ה עוד מקום להמשיך לאכול. אמא הגישה לדני/דנה את המנה השנייה. דני/דנה סיים/ה לאכול וחייך/ה בהנאה. בסיום הארוחה פתח/ה דני/דנה את הארון ואכל/ה שקית שלמה של פריכיות, המון ביסקוויטים עם שוקולד וכוס מיץ. הבטן שלו/ה הייתה כל כך מלאה והוא/והיא הרגיש/ה עייפות. הוא/היא בהחלט לא היה רעב/ה יותר (דירוג ילדים)

## References

[1] Field A.E., Cook N.R., Gillman M.W. (2005). Weight status in childhood as a predictor of becoming overweight or hypertensive in early adulthood. Obes. Res..

[2] WHO WHO Aρnthro Survey Analyser and Other Tools. https://www.who.int/tools/child-growth-standards/software.

[3] NCD Risk Factor Collaboration (NCD-RisC) (2017). Worldwide trends in body-mass index, underweight, overweight, and obesity from 1975 to 2016: A pooled analysis of 2416 population-based measurement studies in 128.9 million children, adolescents, and adults. Lancet.

[4] World Health Organization Obesity and Overweight. https://www.who.int/news-room/fact-sheets/detail/obesity-and-overweight.

[5] Bass R., Eneli I. (2015). Severe childhood obesity: An under-recognised and growing health problem. Postgrad. Med. J..

[6] Chung Y.L., Rhie Y.J. (2021). Severe obesity in children and adolescents: Metabolic effects, assessment, and treatment. J. Obes. Metab. Syndr..

[7] Francis L.A., Susman E.J. (2009). Self-regulation and rapid weight gain in children from age 3 to 12 years. Arch. Pediatr. Adolesc. Med..

[8] Anderson G.H., Hunschede S., Akilen R., Kubant R. (2016). Physiology of food intake control in children. Adv. Nutr..

[9] Mikkilä V., Räsänen L., Raitakari O.T., Pietinen P., Viikari J. (2005). Consistent dietary patterns identified from childhood to adulthood: The cardiovascular risk in Young Finns Study. Br. J. Nutr..

[10] Danielsson P., Kowalski J., Ekblom Ö., Marcus C. (2012). Response of severely obese children and adolescents to behavioral treatment. Arch. Pediatr. Adolesc. Med..

[11] Keller K.L., Assur S.A., Torres M., Lofink H.E., Thornton J.C., Faith M.S., Kissileff H.R. (2006). Potential of an analog scaling device for measuring fullness in children: Development and preliminary testing. Appetite.

[12] Shields B.J., Palermo T.M., Powers J.D., Grewe S.D., Smith G.A. (2003). Predictors of a child’s ability to use a visual analogue scale. Child Care Health Dev..

[13] Wardle J., Guthrie C.A., Sanderson S., Rapoport L. (2001). Development of the Children’s Eating Behaviour Questionnaire. J. Child Psychol. Psychiatry.

[14] Albuquerque G., Lopes C., Durão C., Severo M., Moreira P., Oliveira A. (2018). Dietary patterns at 4 years old: Association with appetite-related eating behaviours in 7 year-old children. Clin. Nutr..

[15] Sinopoulou V., Harrold J., Halford J., Frelut M.L. (2015). Meaning and assessment of satiety in childhood. The ECOG’s eBook on Child and Adolescent Obesity.

[16] Friedman M.I., Ulrich P., Mattes R.D. (1999). A figurative measure of subjective hunger sensations. Appetite.

[17] Triador L., Colin-Ramirez E., Mackenzie M.L., Tomaszewski E., Shah K., Gulayets H., Field C.J., Mager D.R., Haqq A.M. (2021). A two-component pictured-based appetite assessment tool is capable of detecting appetite sensations in younger children: A pilot study. Nutr. Res..

[18] Bennett C., Blissett J. (2014). Measuring hunger and satiety in primary school children. Validation of a new picture rating scale. Appetite.

[19] Toni A.T., Girma T., Hetherington M.M., Gonzales G.B., Forde C.G. (2025). Appetite and childhood malnutrition: A narrative review identifying evidence gaps between clinical practice and research. Appetite.

[20] Adler Institute. https://machon-adler.co.il/en/.

[21] Central Bureau of Statistics Central Bureau of Statistics Home. https://www.cbs.gov.il/en/cbsNewBrand/Pages/default.aspx.

[22] Hammond L., Morello O., Kucab M., Totosy de Zepetnek J.O., Lee J.J., Doheny T., Bellissimo N. (2022). Predictive validity of image-based motivation-to-eat visual analogue scales in normal weight children and adolescents aged 9–14 years. Nutrients.

[23] Monteiro C.A., Cannon G., Levy R.B., Moubarac J.C., Louzada M.L., Rauber F., Khandpur N., Cediel G., Neri D., Martinez-Steele E. (2019). Ultra-processed foods: What they are and how to identify them. Public Health Nutr..

[24] Cooke L.J., Wardle J. (2005). Age and gender differences in children’s food preferences. Br. J. Nutr..

[25] Grzymisławska M., Puch E.A., Zawada A., Grzymisławski M. (2020). Do nutritional behaviors depend on biological sex and cultural gender?. Adv. Clin. Exp. Med..

[26] Cheng Y., Wu X. (2017). The relationship between SES and reading comprehension in Chinese: A mediation model. Front. Psychol..

